# Supplementary material for: Quantum-classical hybrid computation of electron transfer in a cryptochrome protein via VQE-PDFT and multiscale modeling
Source: Chem Sci. 2026 Jan 21;17(7):3492–505. doi: 10.1039/d5sc07528a (PMC12856751; doi:10.1039/d5sc07528a)
Supplement: SC-017-D5SC07528A-s001 [file SC-017-D5SC07528A-s001.pdf]

Supplementary Information (SI) for Chemical Science.
This journal is © The Royal Society of Chemistry 2026

Supplementary Information for: Quantum-Classical Hybrid Computation of Electron Transfer in a Cryptochrome Protein via VQE-PDFT and Multiscale Modeling

December 28, 2025

1 Supplementary Figures

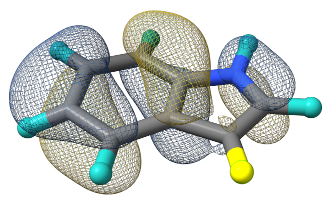

(a) open-shell active orbital 1

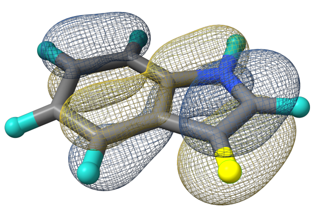

(b) open-shell active orbital 2

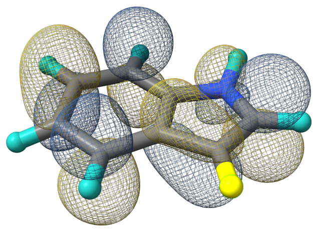

(c) open-shell active orbital 3

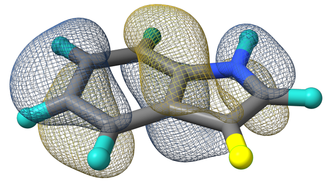

(d) closed-shell active orbital 1

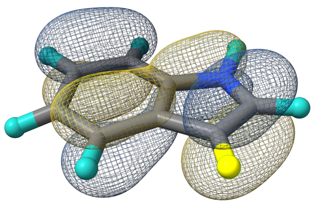

(e) closed-shell active orbital 2

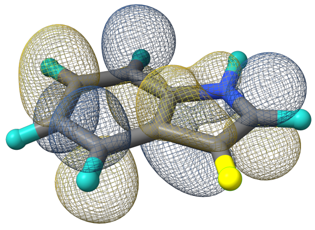

(f) closed-shell active orbital 3

Figure S1: **Active space orbitals visualization of indole rings.** Top row: (3e,3o) open shell active space orbitals; Bottom row: (4e,3o) closed shell active space orbitals. These orbitals are Hartree-Fock canonical orbitals obtained by PySCF's CASCI module [1]. The yellow marked atoms are the hydrogen link-atoms. The Isosurfaces are rendered at value 0.03 a.u. using ChimeraX [2].

2 Supplementary Tables

system	ansatz	active space	qubits	logical circuit				Pauli words (truncated)
				circuit depth	total gates	1-qubit gates	2-qubit gates	
$NH_3 \cdots FCl$	UCCSD	(10e,10o)	20	9164	14079	671	13408	6389
$NH_3 \cdots Cl_2$		(10e,10o)	20	9354	14425	677	13748	6107
$NH_3 \cdots F_2$		(10e,10o)	20	7220	11251	539	10712	6157
$HCN \cdots FCl$		(8e,8o)	16	1592	2628	140	2488	2817
$H_2O \cdots FCl$		(10e,10o)	20	10987	17135	835	16300	12647
$C_2H_2 \cdots FCl$		(10e,10o)	20	3014	4825	217	4608	3727
$C_2H_4 \cdots F_2$		(10e,10o)	20	10949	17085	833	16252	4645
NH_3		(8e,8o)	16	2711	4436	252	4184	2461
HCN		(6e,6o)	12	388	689	45	644	551
H_2O		(8e,8o)	16	1984	3260	180	3080	2305
C_2H_2		(10e,10o)	20	3255	5229	241	4988	4007
C_2H_4		(10e,10o)	20	9344	14633	709	13924	4159
FCl		(2e,2o)	4	9	15	3	12	15
Cl_2		(2e,2o)	4	9	15	3	12	15
F_2		(2e,2o)	4	9	15	3	12	15

Table S1: **Quantum resource summary for calculating dissociation energies of Charge-Transfer dataset.** The first 7 rows are dimers in CT7/04 dataset, while the other rows are monomers of the above dimers. For the largest CT dimer, $C_2H_4 \cdots F_2$, we employed an active space of (10e,10o), which is slightly smaller than the (14e,14o) space used in the original MC-PDFT study, in order to satisfy the limited simulated qubit count within 20 qubits for this proof-of-concept VQE-PDFT demonstration. The reported 'Pauli words' counts correspond to the Pauli decomposition of the CASCI active-space Hamiltonian after applying the default TenCirChem [3] coefficient cutoff (discarding terms with $|c| < 10^{-12}$) prior to fermion-to-qubit mapping (Jordan-Wigner transformation here). Note that all calculations were conducted by noiseless simulation rather than any actual quantum hardware.

(a) **ROUCCSD and HEA resource summary**

**system ErCRY4**	**ansatz**	**active space**	**qubits**	**logical circuit**				**Pauli words**	
				**circuit depth**	**total gates**	**1-qubit gates**	**2-qubit gates**	**truncated**	**total**
open shell	ROUCCSD	(3e,3o)	4	35	55	7	48	118	262
	OHEA	(3e,3o)	4	6	7	2	5	100	219
closed shell	ROUCCSD	(4e,3o)	4	35	56	8	48	118	262
	CHEA	(4e,3o)	4	4	5	2	3	100	219

(b) **HEA resource summary after compiling to a 13-qubit chip**

**system ErCRY4**	**ansatz**	**active space**	**qubits**	**transpiled circuit**				**Commuting groups**	
				**circuit depth**	**total gates**	**1-qubit gates**	**2-qubit gates**	**global (Hamiltonian)**	**local (RDMs)**
open shell	OHEA	(3e,3o)	4	46	61	46	15	26	110
closed shell	CHEA	(4e,3o)	4	18	23	16	7		

Table S2: **Quantum resource summary for the active space of QM region in ErCRY4 protein.** (a) In computation with ROUCCSD, the Pauli words were generated by Jordan-Wigner transformation, while the HEA circuits employed parity transformation with symmetry tapering (two qubits) in the fixed (N_α, N_β) sector, thereby reducing the number of distinct Pauli terms to be measured. The "truncated" denotes Pauli words after the default TenCirChem coefficient cutoff ($|c| < 10^{-12}$), whereas "total" reports the corresponding untruncated term counts. (b) After compilation process, the circuit depth of HEAs increased compared to the logical circuit since the logical gates were decomposed by native gate set. Pauli terms were partitioned into qubit-wise commuting (QWC) groups using the built-in TensorCircuit routine [4], such that any two operators in the same group never act with different non-identity Pauli operators on the same qubit, therefore may be measured simultaneously ($100 \to 26$ global grouping for the truncated Hamiltonian evaluation at each step of VQE). On the other hand, the RDM measurements were performed matrix-element-wise ('total') and thus employ only element-wise QWC grouping ($219 \to 110$ local groups for the full RDM operator set).

Dimer	Methods	E Dimer (Hartree)	E m-1 (Hartree)	E m-2 (Hartree)
$NH_3 \cdots FCl$	VQE	-615.128804	-56.221505	-558.898720
	VQE-PDFT	-616.242235	-56.503348	-559.720469
	MC-PDFT	-616.223810	-56.490022	-559.714003
$NH_3 \cdots Cl_2$	VQE	-975.223380	-56.221467	-918.999406
	VQE-PDFT	-976.527014	-56.503455	-920.015446
	MC-PDFT	-976.507738	-56.490022	-920.010464
$NH_3 \cdots F_2$	VQE	-254.974775	-56.221427	-198.753481
	VQE-PDFT	-255.898334	-56.503449	-199.391897
	MC-PDFT	-255.867282	-56.490022	-199.375496
$HCN \cdots FCl$	VQE	-651.815085	-92.908656	-558.902946
	VQE-PDFT	-653.055775	-93.329282	-559.721513
	MC-PDFT	-653.034226	-93.314369	-559.714003
$H_2O \cdots FCl$	VQE	-634.982061	-76.063217	-558.902897
	VQE-PDFT	-636.098589	-76.371825	-559.721511
	MC-PDFT	-636.070153	-76.349168	-559.714003
$C_2H_2 \cdots FCl$	VQE	-635.759503	-76.850009	-558.902915
	VQE-PDFT	-636.962657	-77.236253	-559.721512
	MC-PDFT	-636.948390	-77.227991	-559.714003
$C_2H_4 \cdots F_2$	VQE	-276.817798	-78.065357	-198.754252
	VQE-PDFT	-277.876723	-78.483346	-199.392029
	MC-PDFT	-277.852830	-78.476804	-199.375496

Table S3: **The absolute energies of dimers and monomers in the Charge-Transfer dataset.** The results were obtained via VQE in CASCI level, VQE-PDFT, and the MC-PDFT method, while the MC-PDFT's results were directly fetched from the Supplementary Material of Ref. [5]. Note that the VQE-PDFT energies are based on CASCI framework, whereas the literature MC-PDFT values rely on CASSCF framework. Because MC-PDFT is not variational with respect to the choice of multiconfigurational reference, these absolute energies are provided for general reference rather than for ranking the methods. For assessing accuracy in dissociation energies, the MUE values reported in Table 1 are more informative. Here, all calculations were performed in the jul-cc-pVTZ basis [6]. The tPBE functional was adopted in both VQE-PDFT and MC-PDFT calculations. The m1 and m2 refer to the two monomers in the m1-m2 dimer, respectively.

**frame**	ΔG^o	λ
157	0.099934408	0.549241928
264	0.043676293	0.347217505
461	0.040885297	0.220829577
631	0.163803428	0.517004701
682	0.038024409	0.495626829
706	0.071676358	0.307290925
777	0.037682775	0.547830361
1077	0.033809862	0.53029316
1107	0.167015044	0.532070337
1237	0.084752646	0.131341481
1243	0.023647298	0.623133927
1311	0.025645922	0.415820148
1368	0.05873396	0.774978721
1502	0.151100112	0.464021283
1603	0.113313698	0.217739833
1626	0.000816062	0.288449438
1627	0.021599893	0.345962916
1965	0.115245417	0.488976157
2071	0.13571487	0.340026962
2180	0.020222078	0.57379004

Table S4: **ΔG^o and λ calculations by empirical HEA on noiseless simulators.** The 20 protein conformations were randomly sampled from the MD simulation of GS ErCRY4 protein in reference [7]. The frame numbers indicate locations of conformations during the entire simulation process. All results are given in eV.

**frame**	H_{DA}	$\lvert H_{DA}\rvert^2$
157	-9.44011E-04	8.91158E-07
264	1.50656E-02	2.26972E-04
461	-3.43295E-02	1.17851E-03
631	-2.49168E-04	6.20846E-08
682	-2.21206E-03	4.89319E-06
706	-1.65424E-04	2.73650E-08
777	-4.04790E-04	1.63855E-07
1077	1.48556E-02	2.20688E-04
1107	-3.80092E-04	1.44470E-07
1237	1.37738E-03	1.89717E-06
1243	1.67444E-03	2.80376E-06
1311	5.25197E-03	2.75831E-05
1368	-1.37476E-03	1.88996E-06
1502	-1.84872E-02	3.41775E-04
1603	3.46027E-03	1.19735E-05
1626	2.59327E-03	6.72507E-06
1627	3.57321E-03	1.27678E-05
1965	-1.51863E-02	2.30625E-04
2071	-2.02746E-03	4.11059E-06
2180	3.41750E-03	1.16793E-05

Table S5: **Electronic coupling calculations.** The 6-31G(d) basis set and the CAM-B3LYP functional were used in all calculations. The 20 conformations were randomly sampled from the MD simulation of GS ErCRY4 protein in reference [7]. The frame numbers indicate locations of conformations during the entire simulation process. All results are given in eV.

**frame**	ΔG^o	λ
157	0.075376004	0.533793409
264	0.066947358	0.550103409
461	0.022934202	0.529776474
631	0.098816411	0.654202407
682	0.102475986	0.552455689
706	0.109047811	0.593777179
777	0.091658923	0.600336874
1077	0.081086545	0.581411027
1107	0.082290769	0.627811309
1237	0.039260208	0.569951056
1243	0.110907047	0.655571383
1311	0.004307911	0.521179077
1368	0.035574098	0.544496837
1502	0.131879075	0.603524833
1603	0.003378448	0.516846946
1626	0.068215042	0.512984809
1627	0.036969471	0.529376022
1965	0.123957956	0.655946507
2071	0.043474286	0.524336485
2180	0.049975887	0.543812624

Table S6: **ΔG^o and λ calculations by ROUCCSD on noiseless simulators.** The 20 protein conformations were randomly sampled from the MD simulation of GS ErCRY4 protein in reference [7]. The frame numbers indicate locations of conformations during the entire simulation process. All results are given in eV.

index	system	shell type	Empirical HEA noiseless	ROUCCSD
1	point-a $TrpB^+$	open-shell	-362.99415	-362.99942
2	point-a $TrpC^0$	closed-shell	-363.26459	-363.26554
3	point-b $TrpB^0$	closed-shell	-363.26777	-363.26682
4	point-b $TrpC^+$	open-shell	-362.98880	-362.98758
5	point-c $TrpB^0$	closed-shell	-363.26994	-363.26920
6	point-c $TrpC^+$	open-shell	-362.99378	-362.99736
7	point-d $TrpB^+$	open-shell	-362.99775	-362.99268
8	point-d $TrpC^0$	closed-shell	-363.26632	-363.26518
λ (eV)			0.34003	0.52434
ΔG^o (eV)			0.13571	0.04347
k_{ET} $(10^8 s^{-1})$			1.89064	2.48679

Table S7: **Single-point energies, Marcus parameters, and the transfer rate of the random conformation.** The conformation was randomly selected as the 2071 frame from the 20 conformations above and calculated within the QM/MM framework. The calculations were conducted by ROUCCSD and empirical HEA on noiseless simulator. The single-point energies are given in Hartree. The 6-31G basis set and the tPBE functional were used in all calculations.

References

[1] Sun Q, Zhang X, Banerjee S, Bao P, Barbry M, Blunt NS, et al. Recent Developments in the PySCF Program Package. The Journal of Chemical Physics. 2020;153(2):024109.

[2] Meng EC, Goddard TD, Pettersen EF, Couch GS, Pearson ZJ, Morris JH, et al. ChimeraX. Protein Science. 2023;32(11):e4792.

[3] Li W, Allcock J, Cheng L, Zhang SX, Chen YQ, Mailoa JP, et al. TenCirChem: An Efficient Quantum Computational Chemistry Package for the NISQ Era. Journal of Chemical Theory and Computation. 2023;19(13):3966-81.

[4] Zhang SX, Allcock J, Wan ZQ, Liu S, Sun J, Yu H, et al. TensorCircuit: A Quantum Software Framework for the NISQ Era. Quantum. 2023;7:912.

[5] Ghosh S, Sonnenberger AL, Hoyer CE, Truhlar DG, Gagliardi L. Multiconfiguration Pair-Density Functional Theory Outperforms Kohn–Sham Density Functional Theory and Multireference Perturbation Theory for Ground-State and Excited-State Charge Transfer. Journal of Chemical Theory and Computation. 2015;11(8):3643-9.

[6] Papajak E, Zheng J, Xu X, Leverentz HR, Truhlar DG. Perspectives on Basis Sets Beautiful: Seasonal Plantings of Diffuse Basis Functions. Journal of Chemical Theory and Computation. 2011;7(10):3027-34.

[7] Xu J, Jarocha LE, Zollitsch T, Konowalczyk M, Henbest KB, Richert S, et al. Magnetic Sensitivity of Cryptochrome 4 from a Migratory Songbird. Nature. 2021;594(7864):535-40.
